# Nanomedicine targeting ECM stiffness: restoring mechanical homeostasis for cancer immunotherapy

**DOI:** 10.1016/j.mtbio.2026.103390

**Published:** 2026-06-23

**Authors:** Mengru Yang, Hanran Jia, Ying Zhang, Xinru Shen, Yongmiao Zhang, Yiting Liu, Xintao Jia, Changxiang Yu, Zhidong Liu

**Affiliations:** a School of Chinese Materia Medica, Tianjin University of Traditional Chinese Medicine, Tianjin, 301617, China; b State Key Laboratory of Chinese Medicine Modernization, Tianjin University of Traditional Chinese Medicine, Tianjin, 301617, China; c Engineering Research Center of Modern Chinese Medicine Discovery and Preparation Technique, Ministry of Education, Tianjin University of Traditional Chinese Medicine, Tianjin, 301617, China; d China-Ghana Belt and Road Joint Laboratory on Traditional Medicine, Tianjin University of Traditional Chinese Medicine, Tianjin, 301617, China

**Keywords:** Extracellular matrix stiffness, Tumor immunosuppression, Nanomedicine, Mechanical homeostasis, Matrix remodeling

## Abstract

The pathological stiffening of the extracellular matrix (ECM) in solid tumors drives immunosuppression and therapeutic resistance. However, non-specific ECM degradation has limited clinical benefit and risks promoting metastasis. To address this dilemma, this review proposes a paradigm shift from indiscriminate physical degradation toward spatiotemporally controlled reconstruction of mechanical homeostasis using smart nanomedicine, an approach that lies at the intersection of materials science, cancer biology, and immunotherapy. We first concisely outline how aberrant ECM stiffness drives a vicious cycle of physical immune exclusion, mechanotransduction-mediated immune reprogramming, and reciprocal fibrotic activation. We then critically analyze how advanced nanomedicine platforms can.

achieve precise ECM modulation without off-target toxicity. Central to this approach is the “mechano-therapeutic window”, an optimal stiffness range that maximizes immune infiltration and therapy sensitization without provoking metastatic risk. Furthermore, we highlight the “priming” strategy, wherein nanomedicine-mediated ECM softening serves as an upstream step to synergistically enhance subsequent immunotherapy, chemotherapy, and radiotherapy, as well as other emerging therapies. Finally, we outline future directions, including the development of adaptive delivery systems that sense and adapt to stiffness dynamics in real-time. By shifting focus from indiscriminate degradation to intelligent mechanical homeostasis, this framework aims to improve cancer immunotherapy outcomes in desmoplastic solid tumors.

## Graphical abstract

**Figure:**
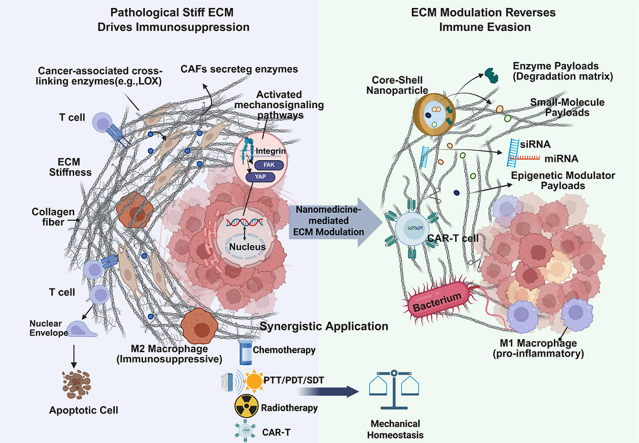

## 1 Introduction

The extracellular matrix (ECM) in solid tumors is not merely a passive scaffold but an active biomechanical player that profoundly influences disease progression and therapeutic response [[1], [2], [3]]. In desmoplastic cancers such as pancreatic cancer, breast cancer, and liver cancer, the ECM can become 5- to 10-fold stiffer than that in normal tissues, driven by excessive collagen deposition and cross-linking mediated by cancer-associated fibroblasts (CAFs) and lysyl oxidase (LOX) family enzymes [[4], [5], [6]]. This pathological ECM stiffening has emerged as a critical physical determinant of immunosuppression and is thought to exclude effector T cells, polarize tumor-associated macrophages (TAMs) toward a pro-tumorigenic M2-like phenotype, and upregulate immune checkpoints via mechanotransduction pathways such as integrin-focal adhesion kinase (FAK), YAP/TAZ signaling, Piezo1, and HIF-1α [[7], [8], [9]].

Given these obstacles, targeting aberrant ECM has been pursued as a promising strategy to enhance therapeutic efficacy. Pharmacological agents including TGF-β inhibitors [10], LOXL2 antibodies (simtuzumab), and hyaluronidases (PEGPH20) [11] have entered clinical trials. However, these approaches have largely failed to demonstrate survival benefits. The reasons are multifaceted: systemic administration risks off-target ECM degradation (i.e., enzymatic breakdown of matrix components) in normal tissues [12]; excessive ECM softening may compromise basement membrane integrity, potentially increasing hematogenous metastasis; and the complex spatiotemporal heterogeneity of ECM remodeling (i.e., alterations in synthesis, cross-linking, and organization) and immune activation makes simple combination regimens ineffective [13,14]. The ultimate therapeutic goal is the restoration of mechanical homeostasis—achieving an optimal ECM stiffness range (the “mechano-therapeutic window”) that maximizes immune infiltration and therapy sensitization without promoting metastasis.

**Scheme 1:**
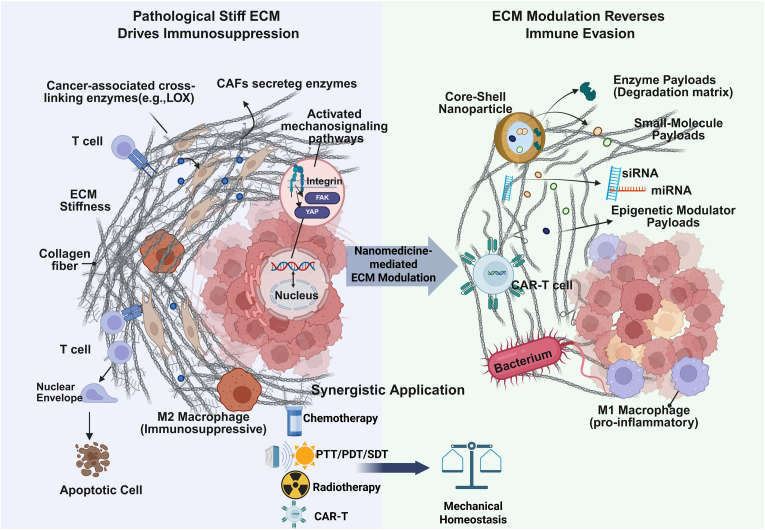
Nanomedicine-Mediated ECM Stiffness Modulation Reverses Tumor Immunosuppression. This Fig. is created with BioRender. com.

This review proposes a paradigm shift from indiscriminate physical ECM degradation (direct enzymatic elimination of matrix barriers) toward intelligent mechanical homeostasis using smart nanomedicine. We argue that the field should move beyond simply “softening” the matrix and instead operate within the mechano-therapeutic window. We also introduce a “priming” strategy, in which nanomedicine-mediated ECM softening serves as an upstream step to synergistically enhance subsequent immunotherapy, chemotherapy, and radiotherapy. We first delineate the multi-scale mechanisms by which ECM stiffness may contribute to immunosuppression. We then critically analyze nanomedicine design strategies that enable precise ECM modulation. Finally, we discuss integration with existing therapies and outline future directions, including defining the mechano-therapeutic window, addressing spatiotemporal heterogeneity of matrix stiffness, and developing closed-loop systems that sense and adapt to stiffness dynamics in real-time (Scheme 1). By bridging materials science, cancer biology, and immunotherapy, this multidisciplinary roadmap aims to engineer mechanical homeostasis as a next-generation cancer treatment strategy.

## 2 Mechanisms of ECM stiffness in driving the immune microenvironment

ECM stiffness serves as a core mechanical factor driving the formation of an immune microenvironment (IME) in tumors [15]. Through a multi-dimensional pathological cascade—encompassing physical immune exclusion, mechanotransduction-driven immune reprogramming, and a self-reinforcing fibrotic-immunosuppressive loop—a stiffened ECM systematically establishes a localized network of immune tolerance [8,16]. This chapter dissects the mechanistic progression of this ECM-stiffness-driven immunosuppressive pathology from an integrated multi-scale perspective.

### 2.1 Physical barrier to immune infiltration

**Fig. 1:**
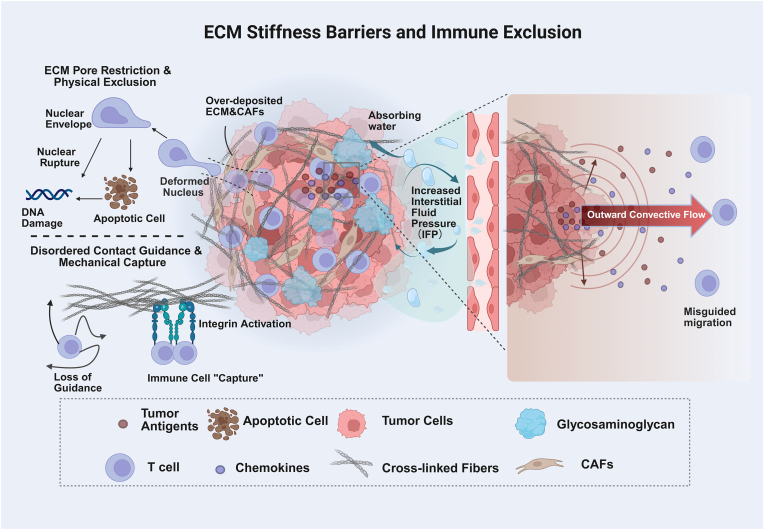
ECM Stiffness Barriers and Immune Exclusion. This Fig. is created with BioRender. com.

A densely cross-linked ECM is believed to impose a formidable physical barrier that restricts immune cell entry into the tumor parenchyma (Fig. 1) [17,18]. Excessive deposition of collagen and glycosaminoglycans reduces matrix pore size, creating steric hindrance that directly impedes T cell penetration [18]. Concurrently, cancer-associated fibroblasts (CAFs) realign collagen fibers into dense anisotropic structures (tumor-associated collagen signature, TACS), which guide T cells to migrate along the tumor periphery rather than infiltrating inward [19]. This spatial segregation constitutes the core biophysical foundation for the “immune exclusion” phenotype observed in malignant tumors [20]. In addition, elevated interstitial fluid pressure (IFP) resulting from glycosaminoglycan swelling and vascular leakage generates outward convective flow that washes away chemotactic gradients, further hindering immune cell trafficking [21]. The physical barrier properties of the ECM are not static but are dynamically determined by the balance between matrix synthesis and degradation. The physical barrier is dynamically maintained by an imbalance between matrix metalloproteinases (MMPs, e.g., MMP-9) and their inhibitor TIMP-1. Persistent mechanical stress upregulates TIMP-1 while suppressing MMP-9 activity via the FAK-ERK pathway, potentially establishing a “mechano-protease” coupling that perpetuates matrix stiffening and reinforces immune exclusion [22].

### 2.2 Mechanotransduction-driven immune reprogramming

Immune cells that successfully breach the peripheral physical barriers and infiltrate the tumor parenchyma remain subject to profound phenotypic regulation imposed by the stiff microenvironment through aberrant mechanotransduction. Mechanical forces are transduced into biochemical signals via transmembrane mechanoreceptors (e.g., integrins and Piezo1) [23], driving the nuclear translocation of transcription factors such as YAP/TAZ, and inducing hypoxia, aberrant expression of immune checkpoints [24,25], and cytoskeletal tension-mediated nuclear deformation, ultimately locking in the exhausted state of immune cells at the chromatin level via mechano-epigenetic remodeling [26,27]. This multi-dimensional mechano-biochemical signaling network, encompassing integrin-FAK-YAP/TAZ, mechanosensitive ion channels, hypoxia, nuclear mechano-epigenetic regulation, and mechano-metabolic coupling, constitutes the core molecular foundation of localized tumor immune tolerance [15,17,28].

#### 2.2.1 Integrin-mediated mechanotransduction and its Immunomodulatory effects

**Fig. 2:**
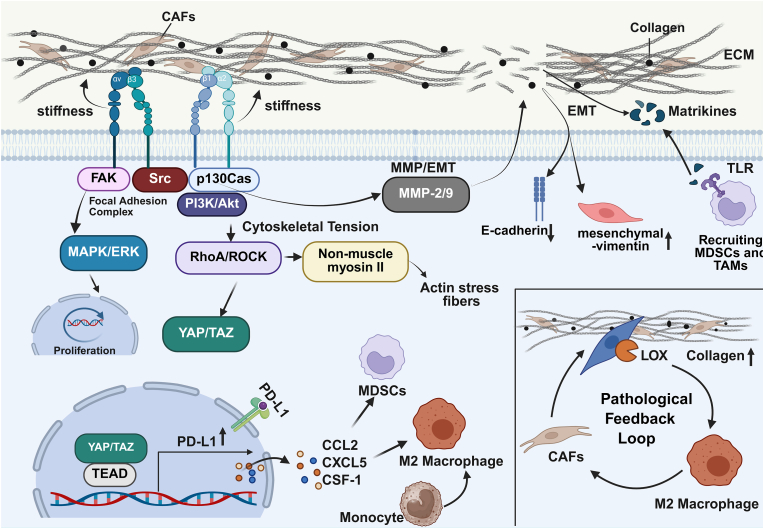
Integrin-FAK Mechanotransduction Activates YAP/TAZ-Mediated Transcriptional Reprogramming, Driving Immunosuppression and Stromal Remodeling. This Fig. is created with BioRender. com.

Integrin serves as the primary transmembrane sensor of ECM stiffness [[29], [30], [31]]. In stiff matrices, elevated stiffness induces conformational activation of integrin subunits such as αvβ3 and α2β1 [32,33], promoting their clustering and the assembly of focal adhesion complexes containing FAK, Src, p130Cas, and PI3K/Akt [34]. This biochemical cascade not only coordinates cellular sensing of the TME's physical parameters but also may influence the malignant progression and immune evasion potential of tumor cells by modulating cytoskeletal tension and gene expression (Fig. 2) [33].

As a central hub in mechanotransduction, the activated FAK signaling axis potentiates tumor aggressiveness through two synergistic pathways [35]. On one hand, FAK promotes proliferation and inhibits apoptosis via the PI3K/Akt and MAPK/ERK pathways [36]. On the other hand, FAK couples with RhoA/ROCK to regulate cytoskeletal tension, establishing a positive feedback loop that amplifies matrix stiffness–cellular tension signaling [37,38]. This cascade upregulates MMP-2/9, which bidirectionally modulate the ECM: they degrade matrix barriers to facilitate tumor cell migration, while simultaneously remodeling the migratory and invasive phenotype of tumor cells by inducing epithelial-mesenchymal transition (EMT) [14]. Concurrently, the extensive ECM degradation generates matrikines—matrix fragments that act as endogenous danger signals. By binding to Toll-like receptors (TLRs) or integrin receptors, these fragments activate local inflammatory responses and recruit myeloid-derived suppressor cells (MDSCs) and TAMs, thereby skewing the immune microenvironment toward an immunosuppressive phenotype [39,40].

Within immune cells, integrin-mediated mechanosensing directly regulates both infiltration efficiency and polarization fate. For effector T cells migrating through dense matrix, integrin signaling modulates actin dynamics to govern nuclear deformation and pore penetration. For cells residing within high-stiffness tumor parenchyma, sustained integrin activation induces profound immunosuppressive phenotypic reprogramming [41]. Studies have shown that aberrant mechanosensing disrupts the assembly integrity of podosomes in dendritic cells (DCs), which may impair their antigen uptake and presentation functions. In macrophages, integrin-triggered signals, often acting through YAP/TAZ, drive polarization toward a pro-tumorigenic M2-like state with elevated IL-10 and TGF-β secretion [42].

These polarized M2-type TAMs, together with persistently activated CAFs, secrete copious amounts of pro-fibrotic factors such as TGF-β in a paracrine manner [43]. This reciprocally drives excessive collagen synthesis and cross-linking, establishing a self-perpetuating mechanobiological loop wherein matrix stiffening, aberrant mechanosensing, and pro-fibrotic activation mutually reinforce one another [43,44].

#### 2.2.2 YAP/TAZ nuclear Translocation-Mediated Transcriptional Reprogramming and immune evasion

YAP/TAZ are the nuclear executors of mechanotransduction (Fig. 2) [45]. In tumor cells, nuclear activation of YAP/TAZ has been implicated in immune evasion. Upon binding to the TEAD family of transcription factors, nuclear YAP/TAZ directly engage the CD274 promoter (encoding PD-L1), upregulating PD-L1 expression on the tumor cell surface at the transcriptional level [46,47]. Concurrently, activated YAP/TAZ drive tumor cells to secrete chemokines (e.g., CCL2, CXCL5) and colony-stimulating factors (e.g., CSF-1), establishing a chemotactic gradient in the tumor microenvironment that specifically recruits MDSCs and induces monocyte differentiation into M2-type TAMs [45,48]. In CAFs, YAP signaling is essential for maintaining their pro-fibrotic phenotype. YAP activation further upregulates matrix cross-linking enzymes such as LOX and collagen, reinforcing the sustained generation of a high-stiffness matrix. Thus, YAP/TAZ function as a central transcriptional hub that translates matrix stiffness into an immunosuppressive network and perpetuates mechanical dysregulation [49].

#### 2.2.3 Mechanosensitive ion channel-mediated calcium dysregulation and immune exhaustion

Mechanosensitive ion channels (Piezo1, TRPV4) provide an ultra-rapid mechanosensing pathway via Ca^2+^ influx [23]. In the pathologically stiff TME, sustained Piezo1 activation and excessive Ca^2+^ influx aberrantly stimulate downstream calcineurin-NFAT signaling, which may drive effector T cells toward an exhausted state [50]. In myeloid-derived cells, Piezo1 signals promote TAM polarization toward a pro-tumorigenic M2-like phenotype by modulating actin dynamics [50]. TRPV4 and other TRP family channels also participate in mechanosensing by regulating Ca^2+^ homeostasis, thereby influencing macrophage inflammatory responses and dendritic cell (DC) maturation [51].

#### 2.2.4 Hypoxic cascade induced by physical compression

Hypoxia resulting from high-stiffness ECM-induced vascular compression drives HIF-1α accumulation [52]. Elevated HIF-1α expression not only upregulates the CD39/CD73-mediated adenosinergic immunosuppressive pathway but also directly binds to the PD-L1 promoter, activating its transcription on tumor cell surfaces [53]. Furthermore, hypoxic signaling recruits Tregs via the secretion of chemokines such as CCL28. Studies have also demonstrated that under hypoxic conditions, HIF-1α stabilization in effector T cells drives excessive glycolysis—initially supporting cytokine production but rapidly exhausting metabolic capacity, triggering irreversible collapse and apoptosis [54]. This fundamentally abrogates their sustained survival and cytotoxic function within the TME.

#### 2.2.5 Nuclear mechano-epigenetic remodeling

Nuclear mechano-epigenetic remodeling is a deeper regulatory layer, in which mechanical forces induce chromatin state remodeling via nucleoskeletal-chromatin coupling [55]. Physical tension transmitted through the Linker of Nucleoskeleton and Cytoskeleton (LINC) complex—composed of nesprins, SUN proteins, and lamins—deforms the nucleus, alters chromatin accessibility, and modulates the activity of epigenetic modifiers such as histone deacetylases (HDACs) and histone acetyltransferases (HATs) [[56], [57], [58]]. Lamin A/C, a key nuclear lamina component, provides mechanical stiffness to the nucleus and mediates force transmission from the cytoskeleton to chromatin [59,60].

Mechanical forces reshape chromatin accessibility within seconds, locking immune cells into an exhausted state [61]. In CD8^+^ T cells, biomechanical stress induces the transcription factor Osr2, which recruits HDAC3 to suppress cytotoxic gene expression and promote terminal exhaustion, suggesting Osr2 as a biomechanical checkpoint [21]. In macrophages, matrix rigidity increases nuclear deformation and H3K9 methylation, potentiating M2 polarization [26]. Collectively, mechanically induced nuclear deformation consolidates T cell exhaustion at the chromatin level, as exhausted CD8^+^ T cells exhibit increased chromatin accessibility at loci encoding inhibitory receptors, including PD-1, TIM-3, and LAG-3 [62].

Mechanical forces exert multi-layered epigenetic control beyond histone modifications, encompassing DNA methylation and non-coding RNA expression [63]. Matrix stiffness modulates DNMT activity, driving promoter hypermethylation of tumor suppressors in cancer cells and reshaping methylation landscapes at immune-related loci within tumor-infiltrating lymphocytes. Concurrently, stiffness-induced mechanosensitive microRNAs (mechano-miRs), such as miR-21 and miR-145, fine-tune fibrotic and immunosuppressive gene networks through post-transcriptional regulation [64]. Deciphering these mechano-epigenetic circuits is essential for developing combination strategies that simultaneously target physical and epigenetic drivers of immunosuppression.

#### 2.2.6 Additional mechanotransduction mechanisms

Beyond the mechanisms discussed above, several other mechanosensitive pathways have recently emerged as critical regulators of tumor immunity.

The MRTF/SRF signalling axis directly couples actin polymerization to gene expression. Upon increased matrix stiffness, enhanced F-actin polymerization sequesters MRTFs away from G-actin, promoting their nuclear translocation and binding to SRF. The MRTF/SRF complex then drives transcription of cytoskeletal and pro-fibrotic genes (e.g., ACTA2, TAGLN) [17,65].

Cytoskeletal tension-mediated metabolic adaptation reprograms immune cell metabolism. In stiff microenvironments, sustained tension activates YAP, which upregulates glycolytic enzymes (HK2, PKM2) and glucose transporters, favouring glycolysis. Conversely, low stiffness promotes oxidative phosphorylation and fatty acid oxidation. This mechano-metabolic coupling affects effector T cells (requiring rapid glycolysis) and regulatory T cells (relying on oxidative metabolism), contributing to T cell exhaustion and Treg enrichment in desmoplastic tumors [66].

Mechanical regulation of the cGAS-STING pathway links stiffness-induced nuclear deformation to innate immune activation. Matrix stiffness-driven nuclear deformation and chromatin compaction cause micronucleus formation and genomic instability, leading to cytosolic double-stranded DNA leakage. This aberrant DNA activates cGAS-STING, triggering type I interferon responses, which can promote antitumor immunity. However, chronic mechanical stress-driven STING signaling may also drive inflammation-mediated immunosuppression and tumor progression [67,68].

### 2.3 The self-reinforcing fibrotic-immunosuppressive loop

The immunosuppressive effects described above coalesce into a self-amplifying loop that sustains both matrix stiffening and immune tolerance. Central to this loop is the reciprocal crosstalk between tumor-associated macrophages (TAMs) and cancer-associated fibroblasts (CAFs) [69].

Within a stiffened ECM, sustained activation of mechanotransduction pathways—including integrin-FAK-RhoA/ROCK and YAP/TAZ signaling—drives infiltrating macrophages toward a pro-tumorigenic M2-like polarization [70]. Recent studies have identified proline-rich tyrosine kinase 2 (PYK2) as a pivotal mechanosignaling integrator in this process. PYK2 consolidates mechanical inputs derived from both integrins and Piezo1, triggering F-actin polymerization and subsequent nuclear translocation, thereby regulating the expression of mechanoresponsive genes such as ACTR3. This concerted regulation dictates monocyte differentiation toward the M2 phenotype [34].

Concomitant with M2 polarization, the soluble factor profile within the microenvironment undergoes marked alteration. M2-type TAMs release abundant immunosuppressive cytokines—including interleukin-10 (IL-10) and transforming growth factor-β (TGF-β)—into the interstitium via paracrine secretion, while upregulating arginase 1 (Arg1) to deplete local L-arginine [71]. Elevated TGF-β not only suppresses effector T cells but also activates CAFs [72]. These activated CAFs, through both Smad-dependent and independent pathways, substantially upregulate the transcription of various collagens and LOXs, thereby exacerbating ECM deposition and cross-linking [73]. This process establishes a self-amplifying pathological cascade within the microenvironment, thereby closing a self-perpetuating loop as the increased ECM density and stiffness further reinforce the mechanical signals that drive initial TAM polarization [43]. In this loop, matrix stiffening promotes M2-type macrophage polarization, M2-derived TGF-β activates CAFs, and CAF-mediated matrix remodeling further elevates ECM stiffness. This cycle not only maintains but progressively amplifies both the physical barrier to immune infiltration and the local immunosuppressive milieu.

**Fig. 3:**
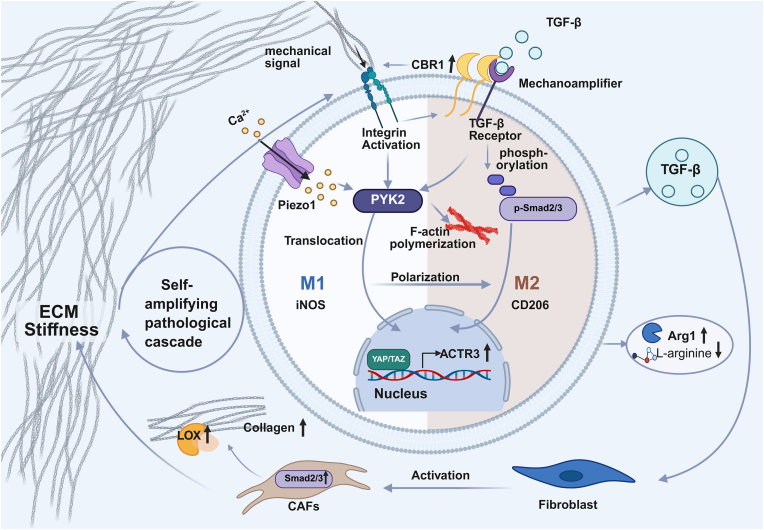
Mechano-Reprogramming of TAMs and Pro-Fibrotic Feedback Loop with CAFs. This Fig. is created with BioRender.com.

Notably, the extent of TGF-β signaling activation is finely modulated by upstream mechanosensors. Emerging evidence indicates that cannabinoid receptor 1 (CBR1) functions as a mechanosensitive co-receptor downstream of integrins, with its expression upregulated within high-stiffness microenvironments. By forming complexes with TGF-β receptors, CBR1 enhances the phosphorylation efficiency of Smad2/3. This “mechano-biochemical” signal integration explains why, even without elevated TGF-β concentrations, its downstream pro-fibrotic effects are significantly amplified within stiffened matrices (Fig. 3).

Two additional immunosuppressive cell types amplify this cycle. First, YAP/TAZ-driven chemokine secretion (CCL2, CXCL5) recruits myeloid-derived suppressor cells (MDSCs), while hypoxia upregulates their PD-L1 expression and arginase activity, depleting L-arginine and suppressing T cells [19,74]. Second, regulatory T cells (Tregs) are enriched via hypoxia-induced CCL28 and TGF-β signaling; the stiff matrix further promotes their oxidative phosphorylation (OXPHOS) metabolism, enhancing suppressive function [75].

Beyond these cellular players, the stiff ECM directly impairs antigen presentation and effector T cell metabolism. Excessive cytoskeletal tension disrupts the microfilament architecture in dendritic cells (DCs), downregulating surface MHC-II and co-stimulatory molecules (CD80, CD86), thereby compromising T cell priming [76]. Concurrently, hypoxia driven by matrix-induced vascular compression stabilizes HIF-1α in cytotoxic T lymphocytes (CTLs), forcing a switch to glycolytic metabolism that rapidly depletes their energetic reserves and triggers activation-induced cell death (AICD) [77,78]. Defective antigen presentation combined with metabolic collapse deprives CTLs and natural killer (NK) cells of the signals needed for sustained proliferation and cytotoxicity.

Additionally, dense matrices impede the diffusion of extracellular vesicles (EVs), trapping tumor-derived exosomes laden with immunosuppressive molecules (PD-L1, TGF-β, oncogenic miRNAs) within the tumor core, thereby amplifying local immune suppression [79]. At the tissue level, stiffness-induced CAF conversion disrupts the formation of tertiary lymphoid structures (TLS) by downregulating CXCL13 and physically compressing lymphocyte aggregation zones, ultimately contributing to an immune-desert phenotype [80].

### 2.4 Dynamic and spatial heterogeneity of ECM stiffness

While the preceding sections delineate how aberrant ECM stiffness drives immunosuppression, matrix stiffness is not a static parameter [81]. Its magnitude and distribution evolve over time and vary across different tumor types as well as across regions within the same tumor, with major implications for nanomedicine-based mechanical interventions [81,82].

Mechanobiological regulation operates across multiple timescales, from rapid integrin/Piezo1-mediated mechanosensing (milliseconds to seconds) and cytoskeletal remodeling (minutes), through transcriptional adaptation (hours), to long-term fibrotic consolidation and mechanical memory (days to weeks) [81]. This multi-scale nature implies that the timing of ECM modulation is as important as its magnitude. Moreover, therapeutic interventions themselves profoundly reshape ECM mechanics [82]. For example, chemotherapy can induce bidirectional remodeling: although tumor debulking may transiently relieve stiffness, therapy-activated CAFs often drive paradoxical collagen cross-linking and secondary stiffening that fosters chemoresistance [82]. Radiotherapy similarly elicits biphasic changes—acute stiffness reduction followed by subacute to chronic fibrotic stiffening via TGF-β-mediated CAF activation, which re-establishes immune exclusion barriers [83]. Beyond these, targeted therapies modulate ECM: anti-angiogenic agents (e.g., bevacizumab) can normalize vascular function and reduce interstitial fluid pressure, indirectly softening the matrix, but prolonged use may paradoxically upregulate CAF-derived collagens [84]. Immunotherapies, such as CAR-T cells, exert mechanical feedback by triggering CAF apoptosis and collagen degradation, yet successful infiltration itself can be limited by the very stiffness they aim to overcome [85]. In addition, endocrine therapies influence ECM cross-linking via LOX family members [86]. These therapy-induced mechanical dynamics create a “mechano-resistant” niche and underscore the need for temporally adaptive nanomedicine strategies rather than single-timepoint matrix softening [[81], [82], [83]].

Beyond its temporal dynamics, ECM stiffness exhibits pronounced spatial heterogeneity within individual tumors. The tumor core typically exhibits the densest, most cross-linked matrix, while the invasive front displays distinct collagen alignment and intermediate stiffness that facilitates invasion [1]. These regional mechanical gradients directly impact immune cell infiltration, drug penetration, and metastatic potential [87]. Consequently, the “mechano-therapeutic window” must be regionally calibrated [87].

Extending beyond intratumoral heterogeneity, the composition and mechanical properties of the ECM vary substantially across tumor types—a distinction that has major implications for personalized mechano-therapies [15]. Pancreatic ductal adenocarcinoma exhibits an exceptionally dense, highly cross-linked matrix (10–100 kPa) that drives T cell exclusion and broad treatment resistance [4]. Triple-negative breast cancer displays a more heterogeneous mechanical landscape, with stiff regions (up to ∼140 kPa by SWE) coexisting with softer, more invasive zones [15]. Other cancers occupy different positions on the stiffness spectrum (e.g., hepatocellular carcinoma: 2–20 kPa; colorectal cancer: stage-dependent from 3 to 14 kPa) [4,6]. These differences dictate that the “mechano-therapeutic window” must be tumor-type-specific, and that nanomedicine strategies should be tailored to the dominant matrix components of each cancer [15].

Collectively, the dynamic and spatially heterogeneous nature of ECM stiffness has motivated the development of feedback-controlled nanoplatforms equipped with real-time sensing and actuation capabilities.

## 3 Design strategies of nanomedicines targeting ECM stiffness

**Fig. 4:**
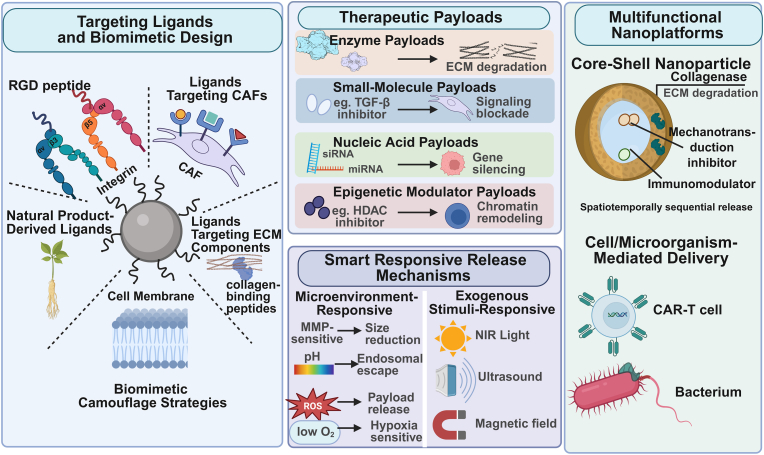
Smart Nanomedicine Design Strategies for Targeting ECM Stiffness. This Fig. is created with BioRender.com.

To guide the design of nanomedicines targeting ECM stiffness, we introduce the concept of a “mechano-therapeutic window”: a stiffness range that balances immune infiltration with metastatic risk. This window is bounded by two biophysical thresholds: an upper limit above which stiffness suppresses immune infiltration, and a lower limit below which ECM softening may promote tumor dissemination. Guided by this quantitative framework, we analyze four interconnected design strategies for nanomedicines: targeted ligand engineering, therapeutic payload selection, smart responsive release mechanisms, and multifunctional synergistic platform construction. These strategies aim to precisely modulate the ECM and restore mechanical homeostasis (Fig. 4).

### 3.1 The mechano-therapeutic window

Based on experimental and clinical data, this article provides a quantitative definition of the “mechano-therapeutic window” [88]. Table 1 lists representative ECM stiffness values for major solid tumors measured by atomic force microscopy (AFM), shear wave elastography (SWE), magnetic resonance elastography (MRE), and transient elastography, etc. The data reveal substantial variation with tumor type and stage, underscoring that the optimal window is tumor-type- and stage-dependent [100].

**Table 1:** Representative ECM stiffness values in human tumors and normal tissues.

Tumor Type/Tissue	Normal/Low Stiffness (kPa)	Tumor/Pathological Stiffness (kPa)	Measurement Technique
Bladder [89]	∼3	Newly diagnosed: ∼8; Recurrent: ∼13	AFM
Breast/mammary tissue [90]	∼28	Invasive ductal carcinoma: 42.5	Multi-modal (including AFM)
Breast/mammary tissue [91]	Benign nodule median: 26.2	Malignant nodule median: 141.6	SWE
Colorectum [92]	0.9	Primary tumor stage: T1: 2.8 T2: 3.5 T3: 8.8 T4: 13.8 Distant metastasis: Present: 13.8 Absent: 7	Venustron system
Glioblastoma [93]	1.5 ± 0.3	1.3 ± 0.3	MRE
Glioblastoma [94]	Gliotic tissue: 0.01–1.8	Low-grade glioma: 0.04–1.4; Glioblastoma: 0.07–1.4	AFM
Liver [95]	—	Low level of malignancy: 8–15 High level of malignancy: 14–18	AFM
Lung [96,97]	2.0 ± 0.1	Idiopathic pulmonary fibrosis: 16.5 ± 2.3	AFM
Pancreas [98]	0.4	1.2	AFM
Thyroid [99]	—	Cutoff for malignant/benign: E-whole-mean 11.36	SWE

Previous studies have established that the upper threshold of the window corresponds to the stiffness above which physical restriction of T cell infiltration and YAP-mediated immunosuppression occur — for instance, roughly 4 kPa in breast cancer models [18,101]. The lower threshold is defined as the stiffness below which excessive matrix softening may compromise basement membrane integrity and increase metastatic risk, with values below approximately 0.5–1 kPa considered potentially hazardous [23]. The optimal range, located between these two boundaries, maximizes immune infiltration and therapeutic sensitization while avoiding pro-metastatic over-softening.

Experimental models provide quantitative support. Tunable PEG-based hydrogels demonstrate that PD-1 suppresses T cell receptor signaling in a stiffness-dependent manner [102]; three-dimensional collagen matrices reveal that increased matrix stiffness inhibits T cell activation via YAP signaling [18]; and low stiffness directs CD4^+^ T cells toward Th1 polarization and promotes M1 macrophages, whereas high stiffness drives Th2 and M2 polarization, fostering a pro-tumor microenvironment [103]. Clinically, SWE offers real-time, non-invasive stiffness quantification (kPa) and correlates with tumor-infiltrating lymphocyte levels and with anti-PD-1 response [104]; MRE provides whole-organ viscoelastic maps with deep penetration [105]. Given the inherent limitations of each technique (e.g., AFM applies only to ex vivo samples; SWE has limited penetration depth for deep-seated tumors and is operator-dependent; MRE requires specialized hardware and offers relatively low spatial resolution), the choice of technique must be guided by the specific research context [106,107]. Despite these limitations, SWE and MRE remain valuable non-invasive tools for longitudinal monitoring. Serial measurements using these modalities can dynamically track changes in matrix stiffness, verify whether stiffness remains within the optimal range, and thereby help avoid the metastatic risk associated with over-softening [106]. On this quantitative foundation, the nanomedicine platforms described in Sections 3.2–3.5 are designed to achieve graded ECM softening within the defined window [108].

### 3.2 Targeting ligands and biomimetic design

The precise distribution of nanomedicines within the tumor interstitium is a prerequisite for achieving effective mechanical modulation. Advanced delivery systems employ active targeting ligands and biomimetic camouflage to achieve specific recognition, enhanced retention, and eventual penetration of the stromal barrier. This section focuses on targeting ligand and biomimetic design strategies. Importantly, these targeting ligands can also modulate the intensity of ECM softening. This is a critical feature for maintaining stiffness within the “mechano-therapeutic window".

#### 3.2.1 RGD peptide modification for integrin targeting

RGD peptide modification, which targets tumor cells and neovasculature, represents one of the most classic active targeting strategies. The RGD sequence specifically recognizes integrins αvβ3 and αvβ5—receptors that are highly expressed on the surface of various tumor cells and tumor-associated endothelial cells [109,110]. RGD-functionalized nanoparticles not only enhance cellular uptake through integrin-mediated endocytosis but also partially interfere with mechanotransduction via competitive binding to integrins, thereby achieving the dual functionality of targeted delivery and signal intervention. It is worth noting that in addition to enhancing uptake, RGD functionalized nanoparticles can also regulate ECM softening by adjusting the coupling strength of integrin ligands. Zhang et al. demonstrated that regulating the coupling strength through DNA strands can reverse persistence [109]. This indicates that RGD-modified nanocarriers can achieve graded mechanical conduction reduction and promote fine-tuning softening within the “mechano-therapeutic window".

#### 3.2.2 Ligands targeting CAFs

As the primary drivers of excessive ECM deposition, CAFs present surface markers—including fibroblast activation protein (FAP), α-smooth muscle actin (α-SMA), and platelet-derived growth factor receptor β (PDGFRβ)—that provide a molecular basis for precise targeting [111]. Among these, FAP is the most promising due to its specific high expression in CAFs and enzymatic activity [111,112]. FAP-targeting antibodies, peptides, and small-molecule inhibitors have been widely employed to construct CAF-specific nanomedicine delivery systems.

Beyond targeting, CAF-directed ligands can tune ECM softening by modulating CAF activation. Mechanistically, CAF-directed ligands deliver anti-fibrotic payloads selectively to CAFs. These payloads — such as TGF-β inhibitors, ROCK inhibitors, or small interfering RNAs — act intracellularly to partially suppress the activated phenotype of CAFs, reducing their expression of α-SMA, collagen I/III, and LOX family enzymes [[113], [114], [115]]. Consequently, ECM deposition and cross-linking are decreased, leading to controlled matrix softening. Importantly, this modulation is dose-dependent and partial, avoiding complete CAF ablation which might disrupt normal stromal homeostasis and inadvertently promote tumor progression [116]. By adjusting ligand density or affinity, researchers can achieve graded reduction in collagen and LOX expression, fine-tuning the extent of ECM softening to remain within the “mechano-therapeutic window” [117]. Additionally, subset-selective ligands (e.g., distinguishing CAF-S1 from CAF-S4) offer further control over softening intensity by targeting only pro-fibrotic CAF subpopulations [118].

#### 3.2.3 Ligands targeting ECM components

Strategies targeting specific ECM components offer an alternative nanomedicine delivery route. Collagen, the most abundant ECM constituent, can be targeted using collagen-binding peptides to anchor nanoparticles within the tumor stroma for long-term retention [119]. The extra domain A (EDA) and extra domain B (EDB) of fibronectin are specifically expressed in tumor neovasculature and stroma, rendering them ideal targets for antibody-drug conjugates and CAR-T cell therapies [120,121]. Importantly, beyond serving as anchoring moieties, these ECM-targeting ligands can directly influence the mechanical outcome by controlling the local concentration and residence time of ECM-modifying payloads [122]. For instance, collagen-binding peptides with tunable affinities enable either sustained, localized softening or shallow, widespread matrix penetration, allowing stiffness reduction to be precisely matched to the mechano-therapeutic window. Furthermore, hyaluronic acid (HA) serves dual functions as a CD44 ligand and a hyaluronidase substrate, creating a self-limiting feedback loop: HA degradation reduces available CD44 binding sites, automatically curbing excessive softening [123,124].

#### 3.2.4 Biomimetic camouflage strategies

Biomimetic nanotechnology based on cell membrane coating, which integrates synthetic polymer cores with the complex lipid bilayer and transmembrane protein networks of natural cell membranes, offers innovative solutions for constructing multifunctional platforms [125]. Coating with membranes derived from erythrocytes, tumor cells, immune cells, or platelets endows nanoparticles with biological properties that enable evasion of mononuclear phagocyte system clearance and active recognition of target cells [126]. In overcoming high-stiffness matrix exclusion, macrophage-coated biomimetic nanoparticles exhibit superior penetration and accumulation due to their natural tropism toward inflammatory microenvironments and preservation of multiple surface receptors [127]. Beyond this passive shielding, biomimetic coatings can actively modulate the intensity of ECM softening by tuning membrane protein composition and density. For instance, nanoparticles coated with CAF membranes derived from different activation states produce graded integrin blocking and mechanotransduction inhibition [128]. Similarly, hybrid membranes combining macrophage and CAF components enable intermediate levels of matrix penetration and softening [129]. This tunability allows stiffness reduction to be precisely confined within the mechano-therapeutic window.

#### 3.2.5 Multifunctional strategies using natural product-derived ligands

Bioactive components from traditional Chinese medicine offer unique advantages as ECM-modulating agents. Quercetin suppresses MMP-2/9 and α-SMA^+^ fibroblasts; Rg3-modified liposomes block integrin αvβ3 mechanotransduction [130,131]; EGCG targets CD44 to inhibit CAF activation [132]. Notably, these natural product ligands enable graded ECM softening via dose- and structure-dependent mechanisms. For instance, low-dose Rg3 mildly blocks integrin clustering, whereas high-dose Rg3 further suppresses YAP nuclear translocation for pronounced softening [133,134]. This tunability allows stiffness reduction to be precisely confined within the mechano-therapeutic window.

### 3.3 Selection of therapeutic payloads

Guided by the mechano-therapeutic window defined in Section 3.1, the selection of therapeutic payloads shifts from “effective degradation” toward window-directed tuning. Below, we analyze four payload categories: matrix-degrading enzymes, small-molecule inhibitors, nucleic acids, and epigenetic modulators.

#### 3.3.1 Matrix-degrading enzyme payloads

Matrix-degrading enzyme payloads achieve rapid disintegration of the physical barrier within tumor tissue through direct hydrolysis of ECM structural components, representing a common approach for reducing ECM stiffness [12]. The most extensively investigated enzymes in this context include hyaluronidase and collagenase, which target hyaluronic acid and collagen, respectively—the two core mechanical components of the ECM [135].

Hyaluronidase specifically degrades excess hyaluronic acid within the TME, reducing interstitial fluid pressure (IFP) and restoring vascular perfusion [136]. Studies have demonstrated that hyaluronidase conjugated to nanoparticle surfaces can progressively hydrolyze and degrade hyaluronic acid within the acidic tumor microenvironment, significantly reducing ECM stiffness and promoting deep penetration of nanomedicines into tumor tissue. The combination of PEGylated recombinant human hyaluronidase PH20 with gemcitabine increases tumor perfusion and vascular permeability in pancreatic cancer models [137]. Collagenase directly hydrolyzes the dense collagen network, creating permeation channels for subsequent therapeutic agents [138].

Enzyme concentration modulation [12,139,140], spatiotemporally responsive delivery [135], and self-limiting designs [141] enable graded control of ECM softening intensity within the mechano-therapeutic window, thereby repurposing matrix-degrading enzymes as precision tools for window-directed mechanical priming. Despite these engineering strategies, intrinsic challenges persist. Enzymes exhibit poor stability and a short half-life in physiological environments, complicating precise control [142]. More importantly, excessive ECM degradation may compromise basement membrane integrity and promote tumor cell metastasis [14]. Therefore, moderate, intensity-controlled ECM softening is sufficient to improve drug penetration and immune cell infiltration, reinforcing the necessity of operating within the optimal therapeutic window.

#### 3.3.2 Small-molecule payloads

Small-molecule inhibitors target pro-fibrotic cascades (TGF-β/Smad, LOXL2, RhoA/ROCK) and offer dose-dependent tunability, a key advantage for graded ECM softening within the mechano-therapeutic window [143]. TGF-β/Smad inhibitors (e.g., SB-505124, galunisertib, pirfenidone) suppress CAF activation and collagen synthesis in a concentration-dependent manner, enabling mild to pronounced softening [140]. LOXL2 inhibitors reduce collagen cross-linking dose-dependently, and when combined with anti-PD-1 therapy, enhance T cell infiltration without over-softening [64]. ROCK inhibitors (Y-27632, fasudil) disrupt mechanotransduction in a concentration-dependent fashion, and nanomedicine delivery allows localized, sustained release for fine-tuned stiffness control [144]. Thus, through nanocarrier-mediated local concentration control, small molecule inhibitors serve as precision tools for window-directed graded softening.

#### 3.3.3 Nucleic acid payloads

Nucleic acid payloads enable dose-dependent silencing of ECM-related genes (TGF-β, CTGF, LOXL2), allowing graded control over softening intensity within the mechano-therapeutic window. By adjusting siRNA dose, release kinetics, or using mismatch-containing sequences, one can achieve mild to pronounced reductions in collagen cross-linking [145]. Beyond direct gene silencing, nanocarrier-mediated delivery of mechanosensitive microRNAs indirectly downregulates integrin-FAK and YAP/TAZ signaling activity, achieving precise intervention in mechanotransduction at the transcriptional level [115]. miR-1246 and miR-17-5p have been shown to regulate integrin-FAK signaling axis activity [146,147]. Unlike irreversible enzyme-based degradation, gene silencing produces slow-onset, reversible, and highly graded softening, which is ideal for maintaining stiffness within the optimal window while avoiding over-softening and metastasis risk [148].

#### 3.3.4 Epigenetic modulator payloads

Addressing the profound mechanisms of epigenetic locking driven by nuclear mechanical deformation discussed previously, the targeted nanomedicine-mediated delivery of epigenetic modulators represents a cutting-edge, subcellular-level intervention strategy in this field. Epigenetic modulators (HDACi, DNMTi) address the deepest layer of mechanical regulation—chromatin compaction driven by nuclear deformation in stiff microenvironments. By restoring transcriptional accessibility of pro-inflammatory genes (e.g., IFN-γ, granzyme B) in exhausted T cells, these agents indirectly contribute to ECM remodeling through enhanced immune-mediated ECM degradation. When delivered via targeted nanomicelles, they enable graded reversal of immunosuppression without directly softening the ECM, complementing other payloads that act on the ECM itself [21].

In summary, the four categories of core payloads described above each possess unique advantages in terms of molecular mechanisms, pharmacokinetic profiles, and spatiotemporal scales of intervention. The rational selection and combination of these payloads, tailored to the temporal sequence of requirements across different pathological steps, constitutes a core strategy for achieving precise modulation of the ECM mechanical microenvironment.

### 3.4 Smart responsive release mechanisms

The unique physicochemical characteristics of the TME provide endogenous or exogenous signals for designing smart responsive nanomedicines. In ECM stiffness modulation, the primary goal is to achieve deep penetration and precise payload release within dense barriers while avoiding off-target disruption of normal tissues [149]. Responsive release mechanisms are particularly suited for operating within the “mechano-therapeutic window”, as they can regulate the degree of ECM softening by controlling the dose, duration, and localization of payload activation.

#### 3.4.1 Microenvironment-responsive nanocarriers

Endogenous stimuli (MMPs, low pH, ROS, hypoxia) enable spatiotemporally controlled release of ECM-modulating payloads, allowing graded degradation, which is a key requirement for confining stiffness within the mechano-therapeutic window. MMP-responsive designs exploit the aberrantly active MMP-2/9 network in desmoplastic tumors; nanoparticles surface-crosslinked with MMP-cleavable substrates shrink upon enzymatic cleavage, releasing smaller cores that penetrate dense collagen networks, and the extent of digestion can be modulated by tuning substrate density or cleavage kinetics [150]. pH-responsive systems leverage the mild acidity of the tumor interstitium and the stronger proton gradient in endosomes/lysosomes [151]; acid-labile bonds or ionizable polymers trigger payload release only after internalization by CAFs or tumor cells, ensuring localized action, with the degree of release adjustable by the density of acid-sensitive groups [152].Redox-responsive nanocarriers utilize elevated glutathione levels to cleave disulfide bonds, enabling dose-dependent softening intensity, while hypoxia-responsive platforms (e.g., azobenzene derivatives) target HIF-1α-rich ischemic cores to deliver matrix-degrading enzymes or immunomodulators to the stiffest fibrotic regions, where the response threshold can be tuned to match the desired softening depth [153]. Collectively, these microenvironment-responsive designs enable graded ECM softening, ensuring that stiffness reduction stays within the mechano-therapeutic window—avoiding both immune exclusion and pro-metastatic over-softening.

#### 3.4.2 Exogenous stimuli-responsive nanocarriers

Exogenous stimuli (light, ultrasound, magnetic fields) enable spatiotemporally controlled ECM modulation with adjustable intensity, complementing endogenous-responsive systems [154]. Photothermal-responsive nanocarriers convert NIR light into localized heat, denaturing collagen fibers and reducing ECM stiffness; the degree of softening can be tuned by varying light intensity or exposure duration, allowing mild to pronounced matrix relaxation within the mechano-therapeutic window [155]. Ultrasound-responsive platforms leverage low-frequency acoustic cavitation to generate microjets and shock waves that dismantle stiff collagen networks, creating low-resistance paths for nanomedicines and immune cells, with the extent of disruption controllable via ultrasound power and duty cycle [156,157]. Magnetic-responsive nanocarriers use superparamagnetic nanoparticles that, under alternating magnetic fields, produce magnetomechanical torque to disrupt ECM scaffolds and interfere with CAF-mediated tension; the softening strength correlates with field strength and frequency, offering precise, non-invasive graded control [158]. Thus, exogenous stimuli-responsive designs enable real-time, controllable ECM softening, confining stiffness within the therapeutic window without over-degradation.

### 3.5 Construction of multifunctional nanoplatforms

Multifunctional nanoplatforms integrate targeting, payload delivery, and responsive release into a single system, enabling temporally sequenced, graded ECM softening within the mechano-therapeutic window. Core–shell and cascade-release designs physically separate payloads into distinct compartments, e.g., an outer shell with matrix-degrading enzymes (collagenase) is released first to lower IFP and create penetration channels, followed by inner-core release of mechanosignaling inhibitors or immunomodulators; the degree of initial softening is controlled by enzyme loading, and subsequent agents fine-tune the mechanical outcome [159]. Biomimetic and cell-mediated delivery (macrophage- or CAF-membrane-coated nanoparticles, bacteria, or CAR-T cells with collagenase nanogels) leverages natural chemotaxis to overcome dense barriers, and softening intensity can be modulated by carrier number or enzyme activity level [160,161].Synergistic co-delivery optimizes drug ratios to address pharmacokinetic mismatches; for instance, co-loading anti-fibrotic drugs (pirfenidone) with immune agonists ensures simultaneous ECM softening and DC maturation, with the extent of softening adjustable by relative dose [162]. Collectively, these multifunctional designs enable spatiotemporally programmable, graded ECM softening that keeps stiffness within the mechano-therapeutic window, forming the basis for combination therapies discussed in Chapter 4.

## 4 Nanomedicine-mediated ECM modulation in combination therapy

The multi-scale pathological cascade driven by ECM stiffness—physical exclusion, mechanotransduction-driven immune suppression, and a self-reinforcing fibrotic loop—creates a hostile microenvironment that severely limits the efficacy of standard cancer treatment. We propose a “priming” strategy: using nanomedicine mediated ECM softening as an upstream step to reshape the mechanical and immune environment of the tumor, thereby making subsequent treatments such as chemotherapy, radiotherapy, or immunotherapy more effective and fully exerting their therapeutic effects in this mechanically optimized microenvironment. This chapter will systematically discuss the strategic integration of ECM regulation mediated by nanomedicine with different therapeutic approaches, with a particular focus on analyzing their synergistic mechanisms, time-sequenced design, and potential for clinical translation (summarized in Table 2).

**Table 2:** Representative studies of nano mediated ECM mechanical remodeling combined with multimodal therapy.

Synergistic Therapy	Design Strategies of Nanoplatforms	ECM targeting mechanism/mechanotransduction pathway	Tumor Type
ICB	Biomimetic hybrid membrane system	FAK targeting (siRNA)	Triple-negative breast cancer [163]
ICB	Thioether hybrid hollow mesoporous organosilica nanoparticles (dsMCu-D@M − Co)	Collagenase mediated collagen degradation + cuproptosis	Pancreatic cancer [164]
ICB + Chemotherapy	Polymeric nanovesicles (Gem/Sul-NP)	Nanovesicles co-deliver the Pin1 inhibitor Sulfopin and gemcitabine to inhibit PSC/CAF activation and reduce ECM deposition	Pancreatic cancer [165]
ICB + Chemotherapy	LOS&FeOX@Gel	TGF-β signaling inhibition (Losartan)	Pancreatic cancer [166]
Chemotherapy	Engineered platelets	Hyaluronidase-mediated HA degradation + TGF-β inhibition	Pancreatic cancer [167]
Chemotherapy	Cal@nChap-CXCL9	CAF deactivation (vitamin D receptor agonist)	Pancreatic cancer [168]
Radiotherapy	RGD-modified liposomes	ROCK pathway inhibition (Y-27632)	Hepatocellular carcinoma [169]
Radiotherapy	a biomimetic Hafnium-based metal-organic framework biomimetic nanoparticles (SHMR), erythrocyte membrane-coated	NO-mediated MMP activation + hypoxia relief	Bilateral tumor model [170]
Radiotherapy	S-MBO@A-RPCM NPs	Deactivation of radiation-activated PSCs	Pancreatic cancer [171]
Radiotherapy	IR-TAM@Alb nanoparticles (albumin nanoparticles)	Metabolic inhibition (OXPHOS) + PD-L1/TGF-β downregulation	Lung cancer [172]
Adoptive Cell Therapy (CAR-T)	"Cell backpack” nanogel	Collagenase-mediated collagen degradation (CAR-T backpack)	Pancreatic cancer [85]
Adoptive Cell Therapy (CAR-T)	In situ synthesized gold nanoreactor	ECM viscosity reprogramming via gold nanoreactor	Solid tumors [173]
Adoptive Cell Therapy (CAR-T)	Heat-inducible CAR-T cells + 3D bioprinted model	Heat inducible CAR T + 3D bioprinted model	Glioblastoma [174]
Adoptive Cell Therapy (T cells)	Redox-responsive supramolecular polyrotaxane nanoparticles (RDPNs@diABZIs)	STING activation + cholesterol depletion	Breast cancer [175]
Photothermal Therapy + ICB	Metal-organic framework (PMOCol)	Collagenase mediated collagen degradation	Pancreatic cancer [176]
Sonodynamic Therapy	Col-H-TiO_2_ NPs	Collagenase mediated ECM degradation (ultrasound responsive)	Pancreatic cancer [177]
Sonodynamic Therapy	FePO@HC	Collagenase mediated ECM degradation (TME responsive)	Pancreatic cancer [178]

### 4.1 Synergistic combination with ICB therapy

**Fig. 5:**
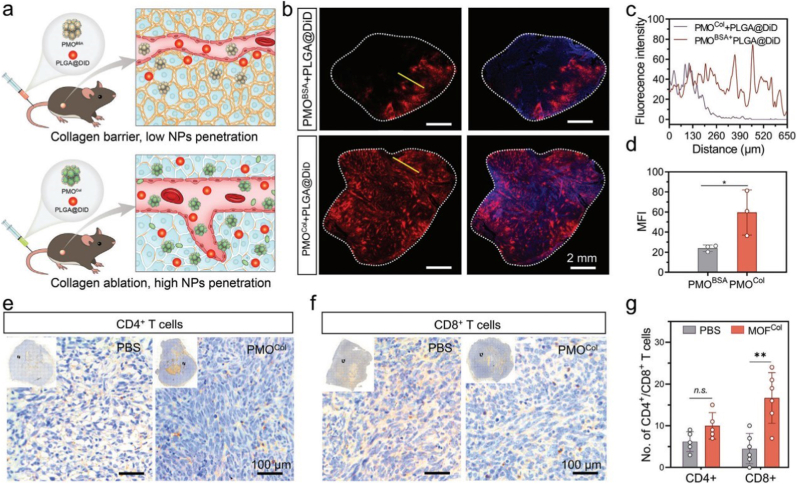
Enhanced NP penetration and immunocyte infiltration after collagen degradation induced by PMOCol. a) Schematic diagram showing the enhanced penetration and accumulation after treatment of PMOCol. Mice were treated with PMOBSA or PMOCol 12 h prior to i.v. injection of PLGA@DiD. After 48 h, tumors were collected for frozen section. b) Fluorescence images of PLGA@DiD distribution in tumor. Scale bar: 2 mm. c) Mean fluorescence intensity (MFI) of DiD in tumor tissues showed in panel (b) (n = 3). d) Fluorescence intensity profiles of DiD along the solid yellow lines in the tumor tissue sections showed in panel (b). Immunohistochemical (IHC) images of CD4^+^ T cell e) and CD8^+^ T cells f) in tumor tissues after in tumor tissues after PMOCol treatment. Scale bar: 100 μm. g) Quantification of CD4^+^ and CD8^+^ T cell amount in panel (e) (n = 5). Data were shown as mean ± SD. ∗p < 0.05, ∗∗p < 0.01. Copyright @ 2024 Small.

The efficacy of ICB therapies, particularly anti-PD-1/PD-L1 antibodies, in desmoplastic solid tumors is severely constrained by the dual barriers of “immune exclusion” and “mechanically-driven immune exhaustion” imposed by the ECM. Nanomedicine-mediated intervention strategies, by targeting and softening the ECM, not only liberate the spatial confinement restricting CD8^+^ T cell infiltration but also disrupt the mechanotransduction- and hypoxia-driven aberrant upregulation of PD-L1 mediated by transcriptional regulators such as YAP/TAZ and HIF-1α. Consequently, the influx of effector T cells and their secretion of IFN-γ remodel the TME toward an inflammation-driven phenotype, effectively converting immunologically “cold” tumors from a state of primary ICB resistance to one of heightened sensitivity. Leveraging this mechanism, several nanoplatforms have demonstrated pronounced synergistic efficacy. For instance, Liu et al. developed a metal-organic framework-based collagenase delivery system (PMOCol) achieving a high loading capacity of 80.3 wt% through optimized payload ratios (Fig. 5) [176]. This system responsively releases collagenase within the TME, effectively degrading excessively deposited collagen fibers in pancreatic cancer tissue and alleviating the physical barrier to T cell infiltration. Concurrently, the co-loaded photothermal agent induces immunogenic cell death (ICD) in tumor cells upon near-infrared irradiation, activating host antitumor immunity. Similarly, an ultrasound-driven biomimetic nanoplatform delivering FAK-targeting siRNA dismantles the physical barrier, reverses M2 TAM polarization, and generates ROS to trigger ICD [163]. Collectively, these studies demonstrate that nanomedicine-mediated ECM priming relieves physical barriers and reshapes the immunosuppressive network, providing a robust foundation for augmenting ICB efficacy.

### 4.2 Combination with chemotherapy

The dense ECM network not only constitutes a physical barrier impeding the deep penetration of small-molecule chemotherapeutic agents but also, through the aberrant mechanotransduction it mediates (e.g., the integrin-FAK pathway), upregulates the expression of multidrug resistance proteins, thereby inducing profound chemoresistance. Precisely softening the ECM via nanomedicine delivery systems enhances the intratumoral penetration efficiency of chemotherapeutic drugs but, more critically, clears the spatial obstacles for DC antigen presentation and T cell infiltration following chemotherapy-induced ICD. This enables a cascade synergy between chemotherapy and endogenous immunity. For instance, Geng et al. developed an engineered platelet-based nanodelivery system (Pts@DOX/HANGs@Gal) co-loaded with doxorubicin, hyaluronidase, and the TGF-β inhibitor galunisertib. Reduction-sensitive release of hyaluronidase in the TME degrades hyaluronan, dismantling physical barriers to drug penetration and immune infiltration. Concurrently, released galunisertib alleviates immune tolerance by inhibiting TGF-β signaling. This dual “ECM degradation and immunosuppression relief” mechanism achieved synergistic enhancement of chemotherapeutic delivery and immune remodeling, significantly potentiating chemo-immunotherapy and reducing metastasis in pancreatic cancer models [167]. Of particular note, Chen et al.’s nanocarrier-based co-delivery platform (Cal@nChap-CXCL9) represents a paradigm shift from traditional “destructive ECM degradation” toward “mechanical homeostasis reconstitution”. This system delivers the vitamin D receptor agonist calcipotriol (Cal), which does not directly hydrolyze pre-existing collagen but instead reverses activated CAFs from a pro-fibrotic to a quiescent state via transcriptional reprogramming, restoring physiological matrix metabolism at its source. This non-destructive cellular “niche reprogramming” dismantles penetration barriers for subsequent gemcitabine delivery and potentiates chemo-immunotherapy efficacy, providing compelling preclinical evidence for abandoning singular physical destruction in favor of systemic microenvironmental homeostasis restoration [168].

### 4.3 Combination with radiotherapy

The microvascular compression and profound hypoxia resulting from ECM stiffness in solid tumors fundamentally undermine the efficacy of radiotherapy at the radiochemical level by limiting the availability of oxygen required for generating lethal ROS. Furthermore, high-dose radiation itself can paradoxically activate CAFs through stress responses, inducing secondary matrix stiffening and contributing to radioresistance. Nanomedicine-based strategies aimed at ECM softening alleviate vascular compression, thereby not only restoring tumor oxygenation to achieve radiosensitization but also effectively disrupting the pro-fibrotic feedback loop activated post-irradiation. Shen et al. developed an RGD-modified liposomal nanoplatform (RGD@LP-Y) encapsulating the ROCK inhibitor Y-27632 (ROCKi), achieving tumor enrichment via targeting of integrin αvβ3. ROCKi-mediated ROCK inhibition significantly downregulated YAP and collagen type I (COL1) expression, reducing ECM density and ECM stiffness, relieving tumor vascular compression, and improving oxygenation status. Concurrently, mechanical unloading drives the M1 polarization of TAMs and promotes DC maturation via the PI3K/AKT/NF-κB pathway [169].

**Fig. 6:**
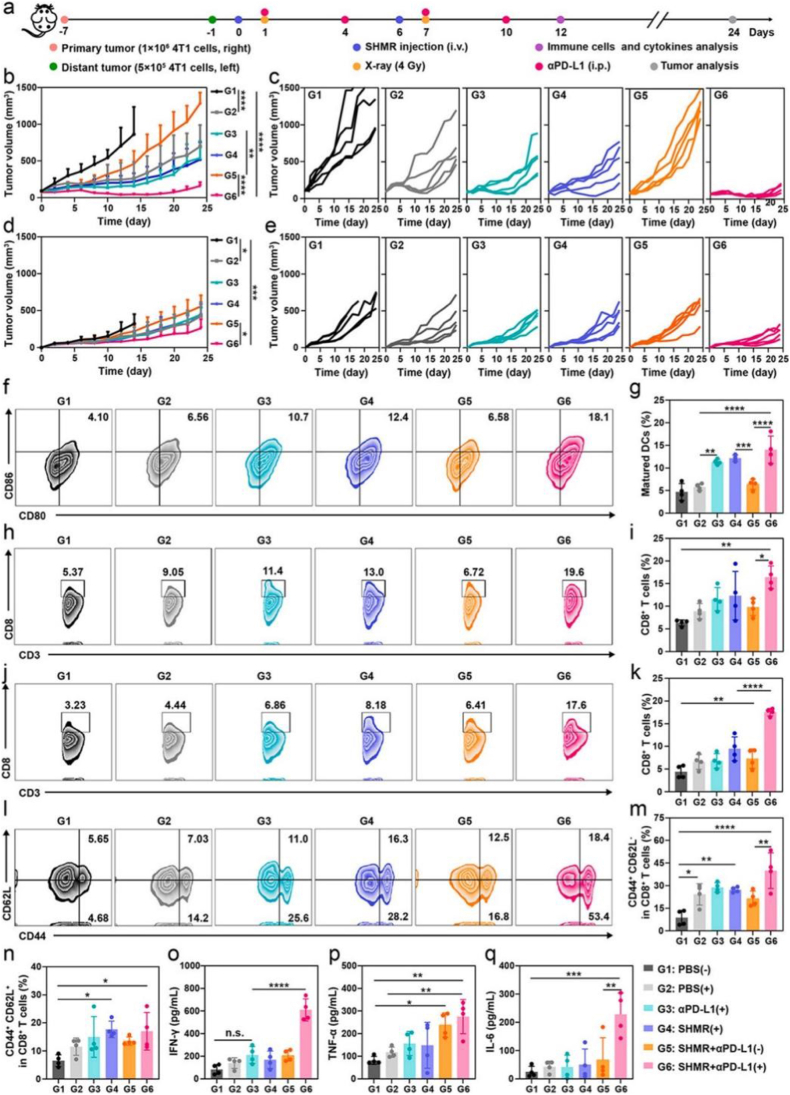
Study of the immunological effects of SHMR-mediated radio-immunotherapy in 4T1 breast tumor-bearing mice. (a) Evaluation of matured DCs (CD11c + CD80^+^CD86^+^) in TDLNs by flow cytometry analysis. (b) Quantitative analysis of DCs in TDLNs (n = 4). (c) Flow cytometric assay of CD8^+^ T cells in tumor tissues. (d) Quantitative analysis of CD8^+^ T cells in tumor tissues (n = 4). (e) Representative flow cytometric plots of Tregs (CD3^+^CD4+Foxp3+) in tumor tissues. (f) Quantitative analysis of Tregs in tumor tissues (n = 4). (g) Representative flow cytometric plots of M1-phenotype (CD11b + F4/80+CD80^+^) macrophages in tumor tissues. (h) Quantitative analysis of M1-phenotype macrophages in tumor tissues (n = 4). (i) Quantitative analysis of M2-phenotype macrophages (CD11b + F4/80+CD206+) in tumor tissues (n = 4). (j-l) Evaluation of the level of IFN-γ (j), TNF-α (k) and IL-6 (l) in tumor tissues by Elisa assay (n = 4). (m) Semiquantitative analysis of the relative HIF-1α area fraction in tumor tissues using Image J (n = 4). Data are represented as mean values ± SD. Significant difference was assessed by one-way ANOVA. ∗P < 0.05; ∗∗P < 0.01; ∗∗∗P < 0.001; ∗∗∗∗P < 0.0001; n.s., no significance. Copyright @ 2024 Nano Today.

Beyond direct intervention in mechanotransduction pathways, indirect remodeling of the immune microenvironment via ECM degradation has also demonstrated potential for synergizing with radiotherapy. Chen et al. designed a biomimetic hafnium-based metal-organic framework (SHMR) with erythrocyte membrane coating for immune evasion, co-loaded with the NO donor sodium nitroprusside (Fig. 6). High-Z hafnium enables X-ray energy deposition for localized radiosensitization. High glutathione in the TME triggers NO release; the resulting peroxynitrite activates MMPs to degrade ECM, dismantling physical barriers to immune infiltration. Released NO also alleviates hypoxia, suppressing HIF-1α-mediated immunosuppression and promoting DC maturation and T cell activation. In bilateral tumor models, SHMR combined with anti-PD-L1 significantly suppressed both primary and distant tumor growth—demonstrating the abscopal effect where localized irradiation induces regression of unirradiated distant tumors [170].

Addressing post-radiotherapy fibrosis, researchers have developed various intervention strategies. Sabu et al. constructed all-trans retinoic acid-loaded nanoparticles (S-MBO@A-RPCM NPs) that, by deactivating radiation-activated pancreatic stellate cells, significantly reduced α-SMA expression and collagen deposition [171]. Zhou et al. developed IR-TAM@Alb nanoparticles that, through inhibition of the OXPHOS pathway, simultaneously downregulated PD-L1 and TGF-β, achieving dual radiosensitization and fibrosis blockade. Collectively, these studies demonstrate that nanomedicine-mediated modulation of ECM stiffness—whether through direct intervention in mechanotransduction or indirectly via ECM degradation and hypoxia relief to remodel the immune microenvironment—provides a robust microenvironmental foundation for radiotherapy-induced ICD and abscopal effects [172]. This multi-layered synergistic strategy, which improves oxygenation through ECM softening while reversing immune tolerance via immune microenvironment remodeling, is emerging as a pivotal direction for overcoming radioresistance in fibrotic solid tumors.

### 4.4 Combination with adoptive cell therapy

**Fig. 7:**
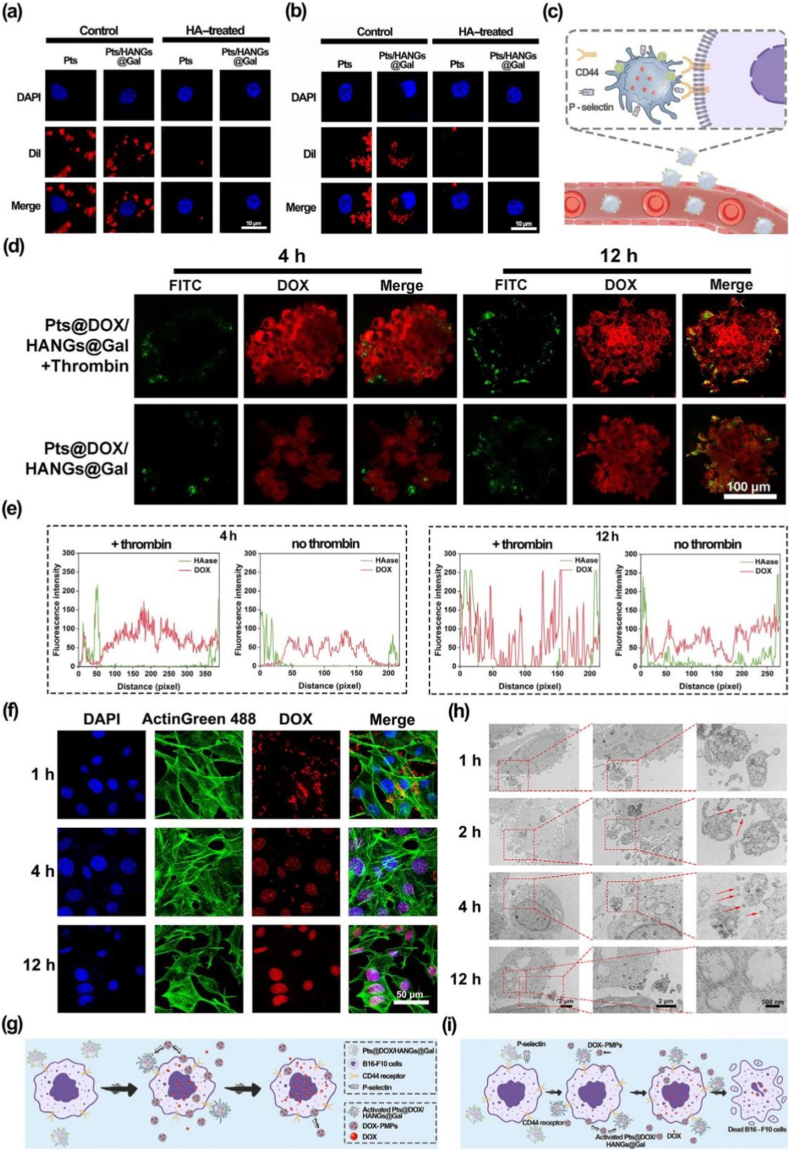
CAR-T cell-bound nanogel backpack delivery system potentiates CAR-T cell therapy in pancreatic cancer. a, Scheme of fabricating the DV1@O-Colase nanogels and the CAR-T cell-bound nanogel backpack delivery system (DV1@O-Colase#CAR-T). b, Schematic illustration of DV1@O-Colase#CAR-T to achieve powerful intratumoural infiltration and localization of CAR-T cells in pancreatic cancer. Copyright @ 2025 nature nanotechnology.

Adoptive cell therapies, particularly CAR-T cells, confront two major challenges in solid tumors: penetrating the dense fibrotic stroma and avoiding rapid exhaustion within the mechano-immunosuppressive network (e.g., inhibitory interactions between dense collagen fibers and LAIR-1 receptors). Nanomedicine-mediated ECM remodeling (including degradation and remodeling), through strategies such as degrading the collagen network or inhibiting CAFs, maximizes the depth of solid tumor infiltration and the durable antitumor efficacy of adoptively transferred cells. For instance, Zhao et al. conjugated collagenase nanogels, modified with CXCR4 antagonist peptides, directly onto CAR-T cell surfaces to form “cellular backpacks” (Fig. 7). During migration toward tumors, CAR-T cells released collagenase in situ, progressively degrading the ECM collagen barrier. Concurrently, CXCR4 antagonism disrupted CXCL12/CXCR4-mediated T cell retention signals. This dual-action strategy increased intratumoral CAR-T cell numbers by 6-fold and significantly prolonged survival in pancreatic cancer models [85]. In a separate approach, Wang et al. employed in situ synthesized gold nanoreactors to modulate ECM viscosity, identifying for the first time ECM viscosity as a physical target for immune evasion. Through viscosity reprogramming, they alleviated the physical barrier posed by the tumor to CAR-T cells, significantly enhancing the suppressive efficacy of adoptive cell therapy against tumor progression and recurrence [173]. Collectively, these studies indicate that achieving ECM mechanical remodeling through diverse approaches—such as cell surface modification and in situ microenvironmental modulation—can effectively dismantle the infiltration barrier confronting adoptively transferred cells and enhance their antitumor efficacy, offering a spectrum of strategies to overcome the bottlenecks of adoptive cell therapy in solid tumors.

### 4.5 Combination with photo/sonodynamic therapy

The efficacy of photo/sonodynamic therapy is critically dependent on both the intratumoral accumulation of energy transducers and adequate tissue oxygen supply. A high-stiffness ECM not only impedes the deep delivery of nano-photo/sonosensitizers but also, through the hypoxic microenvironment it engenders, fundamentally undermines the core therapeutic mechanism— ROS generation. Furthermore, the localized inflammation resulting from photothermal or sonodynamic effects can paradoxically trigger compensatory pro-fibrotic responses. Nanomedicines that concurrently achieve matrix-targeted degradation and hypoxia relief effectively disrupt this vicious “hypoxia-inefficacy-pro-fibrotic” cycle.

Luo et al. constructed collagenase-loaded hollow titanium dioxide nanoparticles (Col-H-TiO_2_ NPs) capable of both responsive collagenase release and sonodynamic therapy upon ultrasound irradiation. ECM degradation significantly reduced tumor interstitial fluid pressure (IFP), creating conditions favorable for deep nanoparticle penetration. Concurrently, ultrasound-triggered ROS generation effectively killed tumor cells and induced ICD, achieving a 65% reduction in tumor volume in pancreatic cancer models [177]. Yin et al. developed a collagenase-loaded nano-sonosensitizer system (FePO@HC) achieving efficient enzyme encapsulation through fusion of human serum albumin with collagenase [178]. Following tumor microenvironment-triggered collagenase release and ECM degradation, this system markedly improved the intratumoral distribution of the sonosensitizer and elevated ROS levels, doubling the tumor-suppressive effect when combined with ultrasound irradiation. Collectively, these findings indicate that nano-enabled ECM stiffness modulation provides a formidable stromal groundwork for photo-/sonodynamic therapy-induced ICD and abscopal effects by abrogating penetration barriers, improving tissue oxygenation, and blocking reactive fibrotic feedback.

## 5 Perspectives

The recognition of ECM stiffness as a major driver of immunosuppression has spurred considerable progress in nanomedicine-based mechanical modulation, yet several conceptual and translational hurdles persist. Moving forward, the field must evolve from empirical, one-dimensional softening toward quantitative, spatiotemporally precise, and ultimately closed-loop strategies that restore mechanical homeostasis.

A key challenge lies in the marked spatiotemporal heterogeneity of ECM stiffness within individual tumors. Stiffness differs between the tumor core and the invasive front, and changes dynamically with disease progression and therapeutic intervention [179]. Conventional bulk analyses average out this complexity. What is needed are integrative imaging and omics platforms that combine second-harmonic generation for collagen imaging, intravital mechanosensing probes, and single-cell or spatial transcriptomics. Such tools could generate a dynamic “mechanical-immune atlas” of each tumor, revealing how local variations in stiffness correlate with the positioning and functional state of immune cells. This, in turn, would enable personalized priming strategies—for instance, delivering matrix-degrading enzymes only to the stiffest regions while sparing areas with normal mechanical properties.

Beyond spatial heterogeneity, clinical translation of stiffness-targeting nanomedicines must confront the hard lessons learned from previous matrix-modulating strategies that failed in the clinic. Two representative examples are PEGPH20 and simtuzumab [180]. PEGPH20 was designed to degrade hyaluronan and improve drug delivery, yet its phase III trial (HALO-301) failed to improve overall survival in pancreatic cancer. Major reasons included systemic toxicities (peripheral edema, muscle cramps, hyponatremia) that forced chemotherapy dose reductions, and the fact that physical barrier removal alone could not overcome the intrinsic biological complexity of the tumor. Simtuzumab failed for a more fundamental mechanistic reason: subsequent studies revealed that the antibody does not inhibit the catalytic activity of LOXL2, nor does it reduce collagen crosslinking or tissue stiffening [181]. Consequently, the clinical trial proceeded without validated target engagement. Taken together, these failures underscore that successful clinical translation must systematically address several interrelated challenges. Patient stratification and biomarker development are critical: tumor stiffness varies widely among individuals, and stratification based solely on hyaluronan expression proved insufficient. Functional and dynamic biomarkers—such as circulating LOXL2 activity, collagen crosslinking products (e.g., pyridinoline), or YAP transcriptional signatures—are urgently needed. Furthermore, the optimal dose and regimen should be defined within the “mechano-therapeutic window”: whereas insufficient softening fails to relieve immune exclusion, excessive softening carries even more dangerous consequences—it can destabilize peritumoral matrix architecture, dismantle physical barriers that confine early-stage tumor cells, release pro-invasive growth factors sequestered in the ECM, and alter integrin-mediated signaling toward a more migratory phenotype, thereby promoting tumor cell invasion, intravasation, and metastatic progression [52,182]. Real-time stiffness monitoring via imaging elastography combined with circulating matrix degradation fragments could enable feedback-controlled adjustment, though such adaptive protocols have not yet been clinically validated. Moreover, long-term safety and off-target effects cannot be overlooked. Systemic ECM modulation may disturb homeostasis in normal organs (e.g., vascular integrity, lung fibrosis resolution), and repeated dosing might provoke rebound fibrosis or immune tolerance—exemplified by the systemic edema observed with PEGPH20 [183]. Finally, the manufacturing and regulatory landscape for smart nanomedicines remains challenging; platforms with multiple responsive components or biomimetic coatings require scalable, reproducible production and rigorous quality control [184,185]. The overarching lesson from these clinical setbacks is that future translation must shift from non-selective matrix degradation to an integrated strategy guided by precision biomarkers, constrained by the mechano-therapeutic window, and balanced with long-term safety and manufacturability.

Looking further ahead, the ultimate ambition is to move from pre-programmed to feedback-controlled nanomedicines. An ideal smart nanoplatform would not only respond to pre-existing tumor microenvironment cues but also sense real-time ECM stiffness changes and adapt drug release accordingly. Achieving this will require integrating highly sensitive mechanical biosensors with adaptive delivery modules. Such feedback-controlled systems could automatically tune ECM softening to stay within the “mechano-therapeutic window”, then release immunomodulators when the physical barrier has been sufficiently lowered. Although speculative, recent advances in synthetic biology, soft robotics, and stimuli-responsive materials are gradually making this feasible.

In summary, the field of mechano-immunology engineering stands at an inflection point. By shifting the focus from indiscriminate ECM degradation to intelligent, homeostasis-restoring interventions(through controlled degradation or remodeling), and by embracing quantitative and feedback-controlled design principles, nanomedicine can unlock the full potential of cancer immunotherapy for desmoplastic solid tumors. The coming decade will likely see the translation of these concepts from proof-of-principle studies into clinically validated priming strategies.

## CRediT authorship contribution statement

**Mengru Yang:** Conceptualization, Writing – original draft. **Hanran Jia:** Investigation, Writing – original draft. **Ying Zhang:** Writing – review & editing. **Xinru Shen:** Formal analysis, Writing – review & editing. **Yongmiao Zhang:** Data curation, Visualization. **Yiting Liu:** Data curation, Investigation. **Xintao Jia:** Formal analysis, Methodology. **Changxiang Yu:** Formal analysis, Validation. **Zhidong Liu:** Resources, Supervision, Writing – review & editing.

## Declaration of competing interest

The authors declare that they have no known competing financial interests or personal relationships that could have appeared to influence the work reported in this paper.

## Data Availability

Data will be made available on request.
